# The geographic mosaic of arms race coevolution is closely matched to prey population structure

**DOI:** 10.1002/evl3.184

**Published:** 2020-06-09

**Authors:** Michael T. J. Hague, Amber N. Stokes, Chris R. Feldman, Edmund D. Brodie, Edmund D. Brodie

**Affiliations:** ^1^ Division of Biological Sciences University of Montana Missoula Montana 59812; ^2^ Department of Biology University of Virginia Charlottesville Virginia 22904; ^3^ Department of Biology California State University Bakersfield California 93311; ^4^ Department of Biology University of Nevada Reno Nevada 89557; ^5^ Department of Biology Utah State University Logan Utah 84322

**Keywords:** Arms race, coevolution, geographic mosaic theory, Na_V_1.4, tetrodotoxin

## Abstract

Reciprocal adaptation is the hallmark of arms race coevolution. Local coadaptation between natural enemies should generate a geographic mosaic pattern where both species have roughly matched abilities across their shared range. However, mosaic variation in ecologically relevant traits can also arise from processes unrelated to reciprocal selection, such as population structure or local environmental conditions. We tested whether these alternative processes can account for trait variation in the geographic mosaic of arms race coevolution between resistant garter snakes (*Thamnophis sirtalis*) and toxic newts (*Taricha granulosa*). We found that predator resistance and prey toxin levels are functionally matched in co‐occurring populations, suggesting that mosaic variation in the armaments of both species results from the local pressures of reciprocal selection. By the same token, phenotypic and genetic variation in snake resistance deviates from neutral expectations of population genetic differentiation, showing a clear signature of adaptation to local toxin levels in newts. Contrastingly, newt toxin levels are best predicted by genetic differentiation among newt populations, and to a lesser extent, by the local environment and snake resistance. Exaggerated armaments suggest that coevolution occurs in certain hotspots, but prey population structure seems to be of particular influence on local phenotypic variation in both species throughout the geographic mosaic. Our results imply that processes other than reciprocal selection, like historical biogeography and environmental pressures, represent an important source of variation in the geographic mosaic of coevolution. Such a pattern supports the role of “trait remixing” in the geographic mosaic theory, the process by which non‐adaptive forces dictate spatial variation in the interactions among species.

**Impact Summary**

Natural enemies often have matched abilities throughout their range, for example, between predator resistance and prey toxins or pathogen virulence and host defenses. This geographic pattern of matched weaponry can arise from coevolution, because counter‐adaptation leads to tightly matched abilities between natural enemies. While coevolution may influence local trait differences, phenotypic variation can also result from other factors, like historical biogeography and local environmental conditions. Common garter snakes (*Thamnophis sirtalis*) evolved resistance to tetrodotoxin (TTX), a deadly neurotoxin found in their prey, the rough‐skinned newt (*Taricha granulosa*). Predator resistance and prey toxin levels are roughly matched throughout the species’ shared range in western North America, a pattern that is generally regarded as evidence of local coadaptation in the arms race. We found evidence that geographic variation in resistance of garter snakes deviated substantially from population genetic structure, supporting the interpretation that local adaptation of predators has occurred. In contrast, toxin levels in prey were clearly predicted by the genetic structure of newt populations and local environmental conditions, two factors unrelated to coadaptation in the arms race. At first glance, matched weaponry between predator and prey seems to be the sole result of intense arms race coevolution, but our results emphasize that landscape patterns of phenotypic variation are determined by a mixture of natural selection, demographic processes, and environmental effects, which together compose the geographic mosaic of coevolution.

Coevolutionary dynamics result from the reciprocal selection generated by ecological interactions among species (Janzen 1980; Thompson 2005). Adaptation and counter‐adaptation occur at the phenotypic interface of coevolution, the traits of each species that mediate their interactions (Brodie and Brodie 1999a; Brodie and Ridenhour 2003). The nature of species interactions and their fitness consequences vary spatially, so heterogeneity of reciprocal selection should generate a geographic mosaic pattern of local coadaptation, in which traits at the phenotypic interface of coevolution tend to covary among locales (Thompson 2005; e.g., Benkman et al. 2003; Zangerl and Berenbaum 2003; Toju et al. 2011). Natural enemies often exhibit matched trait variation across the landscape, for example, in seed traits and their predators (Benkman et al. 2003; Toju et al. 2011) or host and pathogen genotypes (Lively and Dybdahl 2000; Thrall et al. 2002). As such, local coadaptation is often presumed when a pair of interacting species share matched abilities throughout their shared range (Janzen 1980; Brodie and Ridenhour 2003; Thompson 2005; Toju and Sota 2006).

However, other underlying processes can contribute to trait variation in a geographic mosaic of coevolution. Biogeographic history, barriers to dispersal, and population demographics, for example, can structure phenotypic variation across the landscape (Huey et al. 2000; Taylor and McPhail 2000; Langerhans and DeWitt 2004; Keller et al. 2009). As a result, geographic variation in ecologically relevant traits may be influenced by demographic history and population structure in combination with the contemporary effects of reciprocal selection (Thorpe et al. 1995; Alexander et al. 2006; Weese et al. 2012). In the geographic mosaic theory of coevolution, processes like drift and gene flow (termed “trait remixing”) are expected to continually alter the spatial distribution of alleles, potentially interfering with local adaptation (Thompson et al. 2002; Thompson 2005; Gomulkiewicz et al. 2007). Similarly, environmental heterogeneity can contribute to trait variation in the geographic mosaic of coevolution (Toju and Sota 2005; Johnson et al. 2007), particularly when traits are dependent on local conditions (e.g., environmentally derived toxins; Kanoh 1988; Noguchi and Arakawa 2008). Coevolving species are sometimes phenotypically mismatched where traits seem poorly suited for local dynamics (Zangerl and Berenbaum 2003; Toju and Sota 2005; Hanifin et al. 2008), implying that non‐adaptive factors like population structure and the environment are also important sources of variation in the geographic mosaic of coevolution.

In the geographic mosaic of arms race coevolution, common garter snakes (*Thamnophis sirtalis*) evolved escalated resistance to tetrodotoxin (TTX), the deadly neurotoxin found in their prey, rough‐skinned newts (*Taricha granulosa*). Predator and prey have correlated levels of resistance and toxin throughout western North America, which has generally been interpreted as evidence of local coadaptation (Brodie et al. 2002; Williams et al. 2003, 2010; Hanifin et al. 2008). TTX resistance of garter snakes is primarily due to large‐effect mutations in the fourth domain pore‐loop (DIV p‐loop) of the skeletal muscle sodium channel (Na_V_1.4) that disrupt toxin‐binding and confer large increases in resistance (Figure 1) (Geffeney et al. 2002, 2005; Feldman et al. 2010). Variation in snake resistance across the geographic mosaic seems to have evolved in response to selection from toxic prey, because TTX‐resistant alleles occur at high frequency in “hotspots” with highly toxic newts, but are largely absent in surrounding “coldspots” where newts are nontoxic (Brodie et al. 2002; Hanifin et al. 2008; Hague et al. 2017). On the other hand, the underlying basis for TTX production in newts is poorly understood (Hanifin 2010). Newt toxin levels are functionally under‐matched to snake resistance at a number of localities (Hanifin et al. 2008), suggesting that factors other than the arms race may contribute to mosaic variation in newt TTX (also see Hague et al. 2016). Here, we tested whether population structure and local environmental conditions can further explain mosaic variation in the armaments of either species in the arms race.

**Figure 1:**
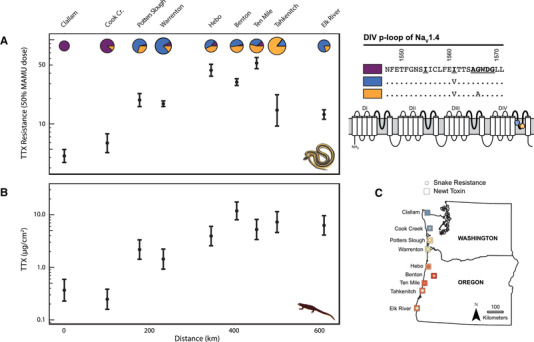
Matching traits in predator and prey suggest local coadaptation in the arms race. (A) Phenotypic TTX resistance of garter snakes (50% MAMU dose) with 95% confidence intervals for populations along the latitudinal transect. The x‐axis represents linear distance (km) from the northernmost sampling site (Clallam; 0 km). The frequency of TTX‐resistant alleles in the Na_V_1.4 channel is shown with pie charts proportional to sample size. To the right, the schematic of Na_V_1.4 shows the four domains of the channel (DI–DIV), with the extracellular pore loops (p‐loops) highlighted with bold lines. Specific amino acid changes in the DIV p‐loop are shown in their relative positions within the pore. The TTX‐sensitive ancestral sequence (purple) is listed, followed by the two derived alleles known to confer increases in channel resistance in this lineage. (**B**) Mean TTX levels of newts (μg/cm^2^) at the same localities along the transect. (**C**) Map inset illustrates population estimates of predator resistance and prey toxins at each location in the geographic mosaic. Blue colors correspond to low estimates of resistance (circles) or TTX (squares), whereas red indicates escalated phenotypes in the arms race.

We conducted fine‐scale population sampling of garter snakes and newts along a latitudinal transect of nine locations on the Pacific Coast in Washington and Oregon (USA) that spans the geographic mosaic (Figure 1; Table S1), ranging from low, ancestral levels of snake resistance and newt toxins (northern Washington) to a hotspot of extreme escalation in both species (central Oregon). We focused our sampling in Washington and Oregon, because other regions of sympatry, particularly California, contain additional resistant garter snakes (e.g., *Th. atratus*) and TTX‐bearing newt species (e.g., *Ta. torosa*) that confound inferences of coevolution between discrete species pairs. At each sampling site in the mosaic, we measured TTX resistance in garter snakes (including whole‐animal phenotypic resistance and Na_V_1.4 channel genotypes) and levels of TTX in newt populations to characterize functional (mis)matches between predator and prey. We then tested whether trait differentiation in the weaponry of each species can be further explained by population structure using genome‐wide single nucleotide polymorphisms (SNPs) and by local environmental conditions using bioclim data (www.worldclim.org). If the geographic mosaic of predator resistance and prey toxins results only from coadaptation in response to a mosaic of reciprocal selection, then trait differentiation of each species should be predicted solely by the local armaments of its natural enemy in the arms race. If mosaic variation in resistance and toxin levels is attributable to processes other than reciprocal selection, then trait differentiation may also be predicted by selectively neutral SNP variation or environmental conditions (Langerhans and DeWitt 2004; Keller et al. 2009).

## Methods

We sampled phenotypic and genomic data from garter snakes (*n* = 169) and newts (*n* = 138) at nine locations along a latitudinal transect in the states of Washington and Oregon (Fig. 1; Table S1) and then compared mosaic patterns of escalation in the arms race to the population genetic structure of each species and local environmental conditions.

### TTX RESISTANCE OF COMMON GARTER SNAKES (*Th. sirtalis*)

We measured phenotypic TTX resistance using a well‐established bioassay of whole animal performance (Brodie and Brodie 1990b; Brodie et al. 2002; Ridenhour et al. 2004; Hague et al. 2017). Briefly, each individual was assayed on a 4 m racetrack to characterize its “baseline” crawl speed, then injected intraperitoneally with a known dose of TTX and assayed for “post‐injection” speed. Population estimates of phenotypic TTX resistance are reported on a scale of mass‐adjusted mouse units (MAMUs) to control for differences in body size (Brodie et al. 2002). Resistance was estimated as the relative performance after injection: the MAMU dose of TTX that reduces performance by 50% of baseline speed. We incorporated racetrack data from previously published estimates of resistance from the same sampling locations to generate precise population estimates of phenotypic resistance in this study (see Table S1; Brodie et al. 2002; Ridenhour 2004).

The 50% MAMU dose was estimated separately for each population from a dose‐response curve using curvilinear regression and the general transform *y*´ = ln(1/*y* – 1) (Brodie et al. 2002). Individuals from each population received a series of TTX doses, with an average of 2.5 different doses per individual. At *y* = 0.5 (i.e., 50%), *y*´ = 0 and the 50% dose is estimable $\hat{x}=\;-\alpha /\beta$ (where α is the intercept and β the slope from the curvilinear regression). Because $\hat{x}$ takes the form of a ratio, the standard error for the estimated 50% dose is calculated using standard methods for the variance of a ratio (Lynch et al. 1998 p. 818; Brodie et al. 2002). Confidence intervals of 95% were calculated as ±1.96 SE. Regression was performed in R with the “lmer” function implemented in the lme4 package (Bates et al. 2015). The individual ID of each snake was included as a random effect to account for the fact that each snake received multiple injections. Distribution and leverage analysis indicated that a transformation of the *x* variable (MAMU of TTX) was needed, so we transformed the data using *x*´ = ln(*x* + 1) (Brodie et al. 2002). Differences among populations in phenotypic TTX resistance were deemed significant if 95% confidence intervals did not overlap by more than half of a one‐sided error bar (Cumming and Finch 2005). The transformed MAMU values were used in the subsequent regression and cline‐fitting analyses of TTX resistance along the transect (see below).

For each snake, we also evaluated the amino acid sequence in the DIV p‐loop of the Na_V_1.4 channel. Methods for Sanger sequencing are described in Hague et al. (2017). A 666 bp fragment that includes the DIV p‐loop region of Na_V_1.4 was sequenced for each individual as part of a recent analysis demonstrating that the gene encoding Na_V_1.4 (*SCN4A)* is located on the Z sex chromosome of *Th. sirtalis* (Gendreau et al. 2020). Colubrid snakes, including garter snakes, have heteromorphic sex chromosomes (ZZ males, ZW females) that are non‐recombining (Vicoso et al. 2013; Augstenová et al. 2018), and females are hemizygous for the Z‐linked *SCN4A* gene. The haplotype phases of DIV p‐loop sequences from homogametic males were inferred computationally with the program PHASE (Stephens et al. 2001; Gendreau et al. 2020). The translated DIV p‐loop coding sequences were then tested for departures from Hardy–Weinberg Equilibrium (HWE) using a joint test for HWE and equality of allele frequencies (EAF) using the *HWTriExact* function in the R package HardyWeinberg, which accounts for the hemizygous sex (Graffelman and Morales‐Camarena 2008; Graffelman and Weir 2018a,b; Gendreau et al. 2020).

We used the Na_V_1.4 haplotype data from Gendreau et al. (2020) to calculate pairwise F_ST_ differentiation at the DIV p‐loop in the program Arlequin (Excoffier and Lischer 2010) and used multiple regression of distance matrices (MRMs; see below) to test for a relationship between F_ST_ differentiation in the DIV p‐loop and phenotypic differentiation in whole‐animal TTX resistance. Importantly, our analysis here of the DIV p‐loop does not account for other unknown factors that also contribute to variation in whole‐animal resistance (Avila 2015; Feldman et al. 2016). Estimates of TTX resistance from individual snakes in the Pacific Northwest tend to be normally distributed within any given population of *Th. sirtalis* (Brodie and Brodie 1999a, 1990b), indicating that a discrete polymorphism in the DIV p‐loop does not solely explain variation in phenotypic TTX resistance.

### TTX LEVELS OF ROUGH‐SKINNED NEWTS (*Ta. granulosa*)

We estimated levels of TTX in newts using a Competitive Inhibition Enzymatic Immunoassay (CIEIA) and TTX‐specific antibodies (Gall et al. 2011; Stokes et al. 2012). We quantified the amount of TTX in a 5 mm circular skin punch from the dorsum of each newt using a human skin‐biopsy punch (Acu‐Punche, Acuderm Inc.; Hanifin et al. 2004, 2002; Hague et al. 2016). These data were used to estimate the dorsal skin concentration of TTX (μg/cm^2^) in each individual. TTX is uniformly distributed throughout the dorsum and levels of TTX in the dorsal skin are tightly correlated with toxicity in other regions (Hanifin et al. 2004). We conducted a two‐way ANOVA to test whether TTX differed by population and by sex, because past work suggests toxin levels may vary by sex (Hanifin et al. 2002). Distribution and leverage analyses indicated that a *x*´ = log(*x* + 0.1) transformation of TTX was needed. The transformed TTX values were used in the subsequent regression and cline‐fitting analyses of TTX along the transect (see below).

### FUNCTIONAL ANALYSIS OF TRAIT MATCHING

Following Hanifin et al. (2008), we estimated functional levels of snake resistance and newt TTX to visualize whether predator and prey exhibit matched levels of escalation along the transect. The model provides a rough estimate of functional interactions between snake resistance and newt TTX based on an extensive body of work (Brodie and Brodie 1991, 1990b; Hanifin et al. 2004, 1999; Brodie et al. 2002; Williams et al. 2002; Brodie and Ridenhour 2003; Ridenhour et al. 2004). Localities are considered “matched” if a sympatric interaction between predator and prey could potentially result in variable fitness outcomes for both species, leading to reciprocal selection between snake resistance and newt TTX (Hanifin et al. 2008). For each locality, we inferred whole‐newt levels of TTX (mg) and the dose of TTX (mg) required to reduce the performance of co‐occurring snakes to 15, 50, and 85% of their baseline performance. The TTX dose required to reduce snake performance by 50% is considered a perfect functional match between newt TTX and snake resistance. The 15 and 85% doses delimit the range of functionally relevant doses for snakes. At performance levels <15%, all snakes that ingest newts are fully immobilized or killed and newts escape, whereas at performance levels >85% all snakes are unaffected and captured newts die (Williams et al. 2003, 2010; Hanifin et al. 2008). Localities where the full range of TTX doses found in newts fall outside the 15–85% region of phenotypic space are considered phenotypic “mismatches,” such that variable fitness outcomes and reciprocal selection are unlikely to occur.

Methods for estimating functionally comparable values of newt TTX and snake resistance are described in Hanifin et al. (2008). Briefly, we extrapolated our measures of newt TTX in skin samples (μg/cm^2^) to the whole animal (mg of TTX/newt) using standard methods. For snakes, the 15, 50, and 85% MAMU doses were extracted from the dose‐response curves of each population. As described in Hanifin et al. (2008), we converted snake resistance from MAMUs based on intraperitoneal (IP) injections to milligrams of TTX in oral doses using the following equation:

$$\mathrm{IPdose}\left(\mathrm{mg}\right)=(\theta \times 0.00001429)\times \mathrm{snake}\;\mathrm{mass}\left(g\right)$$

where θ is the MAMU dose and 0.0001429 is the conversion factor (1 MAMU = 0.01429 μg TTX per gram of snake). The effects of TTX are dependent on body size, so we estimated the oral dose of TTX required to slow the average adult snake (mean adult mass = 52.84 g). We then converted the IP dose (mg) to the oral dose required to achieve the same performance reduction by multiplying the IP dose by 40. Estimates of orally ingested doses of TTX (mg) required to reduce snake performance by 15, 50, and 85% were used as a functionally equivalent metric to compare predator phenotypes to those of the prey (Table S2).

### ddRADseq LIBRARY PREPARATION AND SEQUENCING

We generated SNP data from double digest restriction‐site associated DNA sequenced (ddRADseq) to characterize the population structure of predator and prey, which served as a neutral expectation for trait differentiation in the geographic mosaic of coevolution. We prepared ddRADseq libraries separately for garter snakes and newts using the protocol described in Peterson et al. (2012). Genomic DNA was extracted using the DNeasy Blood & Tissue kit (Qiagen Inc., Valencia, CA.). We digested 600 ng of genomic DNA for each sample using the restriction enzymes *MfeI* and *SbfI* for snakes and *EcoRI* and *SbfI* for newts. Unique combinations of individual P1 and P2 barcoded adapters were annealed to the digested genomic DNA of each sample. Each barcode was six base pairs long and differed by at least two nucleotides. After barcoding, snake and newt samples were pooled separately, purified with AmpureXP beads (Beckman Coulter, Inc., Brea, CA, USA), and size selected for 500–600 bp fragments using a Pippin Prep (Sage Science, Inc., Beverly, MA, USA). We enriched the adapter‐ligated fragments in the size‐selected libraries using 16 PCR cycles and then purified the product with AmpureXP beads. The snake and newt libraries were each run on two lanes of the Illumina HiSeq 2500 platform (Illumina, Inc., San Diego, CA, USA) to generate 125 bp paired‐end reads.

Read quality of the raw sequence data was assessed using FastQC 0.11.5 (Andrew 2016). We used *process_radtags* in Stacks 1.46 (Catchen et al. 2013) on both the snake and newt datasets to de‐multiplex reads and remove sequences with low‐quality scores or uncalled bases. The garter snake reads were aligned to the *Th. sirtalis* genome using Bowtie2 2.2.9 (Langmead and Salzberg 2012). We discarded reads that did not align or had more than one match to the genome. We used *ref_map.pl* in Stacks to assemble the reference‐aligned sequences into loci, with a minimum depth of three (‐*m*). For the newt dataset, we used the *denovo_map.pl* pipeline in Stacks, because a reference genome is not currently available. We used a minimum depth of three (‐*m*), a distance of three between stacks (‐*M*), and a distance of three between catalog loci (‐*n*). For both species, we used *populations* in Stacks to select loci with a minimum depth of 10× coverage. To avoid linkage among sites within the same locus, we only retained one SNP per locus.

We used the dartR package in R (Gruber et al. 2017; R Core Team 2018) to remove loci and individuals with >30% missing data. We also removed loci with a minor allele frequency (MAF) <5%, including those that were invariant. Finally, we removed putative loci under selection. The program BayeScan 2.1 was used to search for loci with F_ST_ coefficients that were significantly different than those expected under neutrality (Foll and Gaggiotti 2008). The Bayesian analysis used 20 pilot runs with 5000 iterations followed by an additional burn‐in of 50,000 and 50,000 output iterations. An outlier analysis with FDR‐corrected *p*‐values (*q*‐values) < 0.05 was used to identify and remove outlier loci putatively under selection.

Illumina HiSeq sequencing yielded an average of 2,451,623 raw reads per sample (*n* = 143) for garter snakes. The *ref_map* pipeline identified an average of 13,501 loci per individual, with a mean of 80× coverage. After additional filtering, we retained 1,027 unlinked neutral SNPs in 132 individuals. For newts, sequencing produced an average of 1,093,529 raw reads per sample (*n* = 137). After initial quality control in *process_radtags*, the *denovo_map* pipeline in Stacks identified an average of 32,469 loci per individual, with a mean of 19x coverage. After further filtering steps in *populations*, dartR, and Bayescan, we retained 3634 unlinked neutral SNPs in 123 individuals. The final datasets comprised a larger number of SNPs for newts (3634) than snakes (1027), so we reran the subsequent analyses with a random subsample of 1027 SNPs for newts to generate equivalent datasets for each species. Results from the subsample were highly similar to the full dataset, so only results from the full analysis are presented herein.

### ANALYSIS OF GEOGRAPHIC POPULATION STRUCTURE

Using the filtered SNPs for each species, we calculated average observed heterozygosity (H_O_) and expected heterozygosity (H_E_, also referred to as gene diversity [H_S_]) for each population using the hierfstat package in R (Nei 1987; Goudet and Jombart 2015). We also calculated nucleotide diversity (π) for each population using the concatenated set of SNPs in Arlequin (Nei 1987; Excoffier and Lischer 2010). Estimates of within‐population genetic diversity from the final SNP datasets of each species are reported in Table S3. To estimate neutral population genetic differentiation, we calculated global and pairwise F_ST_ values (Weir and Cockerham 1984). Confidence intervals were estimated by running 1000 bootstraps over loci using the hierfstat and stAMPP packages in R (Pembleton et al. 2013). Estimates of pairwise F_ST_ are reported in Table S4.

We tested for a pattern of isolation‐by‐distance (IBD) along the transect by performing Mantel tests on matrices of linearized pairwise F_ST_ and geographic distance (Rousset 1997). We also conducted distance‐based redundancy analyses (dbRDA), which are thought to be more reliable than Mantel tests at detecting spatial patterns like IBD (Legendre and Fortin 2010; Meirmans 2015). We conducted dbRDA analyses in the vegan package in R (Oksanen et al. 2019) to test for a relationship between pairwise F_ST_ values and the geographic coordinates (latitude and longitude) of sampling locations. We assessed the significance of IBD tests with 1000 permutations (Fig. S1).

Pairwise F_ST_ is a relative measure of among‐population differentiation that is inherently dependent on levels of within‐population diversity (Charlesworth 1998; Cruickshank and Hahn 2014). Therefore, we also assessed the population structure of each species using a principal coordinate analysis (PCoA) in the dartR package in R. The first axis (PCo1) from the PCoA captured latitudinal variation along the transect for both snakes and newts (Fig. 3), so we used the PCo1 values for each individual as a neutral expectation of population structure in our cline‐fitting analyses (see below). We also assessed population structure using a Bayesian assignment approach implemented in the program STRUCTURE (Pritchard et al. 2000; Falush et al. 2003). We estimated the optimal number of genetic clusters (K) ranging for one to nine, without populations included as priors. The model assumed population admixture and correlated allele frequencies (Falush et al. 2003). The analysis first ran for 100,000 iterations as burn‐in and then we collected data from the following 1,000,000 interactions in 10 different independent runs. STRUCTURE HARVESTER (Earl and vonHoldt 2012) was used to detect the most probable K using the Evanno's method (Evanno et al. 2005). Ancestry proportion (Q) values of the 10 runs for each value of the most probable K we averaged using CLUMPP (Jakobsson and Rosenberg 2007) and visualized using the pophelper package in R (Francis 2017). We calculated the average Q value for each population, which represents the fraction of membership to each genetic cluster (K). These Q estimates were also used as a neutral expectation in cline‐fitting analyses (see below) and compared to the cline results of PCo1 from the PCoA.

### ANALYSIS OF LOCAL ENVIRONMENTAL DATA

We also sought to evaluate environmental contributions to trait variation in the geographic mosaic of coevolution. We extracted temperature and precipitation bioclim data (www.worldclim.org) for each locality along the transect. These environmental variables should be especially important for ectotherms and are considered principal determinants of the plant communities that define habitat types (i.e., biomes, ecoregions, etc.). We summarized variation in the 19 bioclim variables using a principal component analysis (PCA). The first two axes explained 89.2% of the variation, which we selected as a summary of local environmental conditions along the transect. PC1 generally captured latitudinal variation in annual mean temperature and precipitation, with positive values indicating warm, dry conditions and negative values comprising colder, wet conditions. PC2 captured temporal fluctuations in temperature, with positive values representing greater seasonality and annual temperature range and negative values comprising less annual variation in temperature (Table S5).

We used environmental PC1 and 2 from the bioclim data to test for a pattern of isolation‐by‐environment (IBE), which is characterized by a correlation between genetic differentiation and environmental distance, for example, because migrants are selected against in foreign environments (Nosil et al. 2009; Wang and Bradburd 2014). As in our tests of IBD, we used a dbRDA to test for IBE characterized by a relationship between pairwise F_ST_ and environmental PC1 and 2. We also used a conditional dbRDA to test for the presence of IBE after removing the effect of latitude and longitude on F_ST_ (i.e., IBD).

### INFERRING CONTRIBUTIONS OF POPULATION STRUCTURE AND THE ENVIRONMENT TO THE GEOGRAPHIC MOSAIC OF COEVOLUTION

Using our phenotypic, genetic, and environmental data, we tested whether a focal trait in the arms race is predicted by (1) escalation in the armaments of the natural enemy, (2) population structure of the focal species, and (3) local environmental conditions. Multiple regression of distance matrices (MRMs) is an extension of Mantel tests that involve multiple regression of a response distance matrix on explanatory matrices (Manly 1986; Smouse et al. 1986; Legendre et al. 1994; Lichstein 2007). Partial regression coefficients can be used to understand the relationship between two matrices while controlling for the effects of a third matrix (Rosenblum 2006; Toju et al. 2011). For each species, we generated three distance matrices: (1) pairwise phenotypic differentiation in the coevolutionary trait (e.g., TTX resistance of snakes), (2) pairwise genetic differentiation from neutral SNPs (F_ST_), and (3) pairwise differences in environmental conditions (environmental PC1 and 2). We then tested whether population patterns of phenotypic escalation in a focal species (e.g., TTX resistance of snake) are explained by trait escalation in the natural enemy (TTX of newts), population genetic differentiation (pairwise F_ST_ of snakes), and local environmental conditions (environmental PC1 and 2). All MRM analyses were conducted using the “MRM” function in the R package ecodist (Goslee and Urban 2007).

Our goal was to dissect how population structure and local environmental conditions each contribute to trait variation in the geographic mosaic; however, the analysis was complicated by multicollinearity among our latitudinal datasets. The genomic analyses revealed a tight relationship between genetic differentiation (pairwise F_ST_) and geographic distance along our latitudinal transect, indicative of strong IBD in both species (Figs. S1 and S2). Geographic distance is often also spatially correlated with environmental conditions (Nosil et al. 2009; Bradburd et al. 2013; Wang and Bradburd 2014) and, along our latitudinal transect, geographic distance was strongly correlated with environmental PC1 from the bioclim data (Mantel test: *r* = 0.400, *p* = 0.033). The strong pattern of IBD along the latitudinal transect made it difficult to disentangle how genetic differentiation and the environment each contribute to variation in coevolutionary traits. This multicollinearity problem is illustrated by the correlations among newt TTX levels, newt pairwise F_ST_, geographic distance, and environmental PC1 (Fig. S2).

To address issues of multicollinearity, we complemented MRM analyses with conditional redundancy analyses (RDAs), which have been proposed as more appropriate for analyses of spatial data (Legendre and Fortin 2010; Legendre et al. 2011; Holding et al. 2018). Partial Mantel tests and MRMs can be misleading in the presence of autocorrelation (Raufaste and Rousset 2001; Rousset 2002; Guillot and Rousset 2013; Legendre et al. 2015), in addition to other reported problems with inflated type I error rates (Balkenhol et al. 2009; Guillot and Rousset 2013) and low power (Legendre and Fortin 2010; Graves et al. 2013). We conducted a similar set of RDA analyses and calculated the proportion of variance in coevolutionary traits explained by each explanatory variable. Because RDAs do not rely on dissimilarity measures (i.e., pairwise F_ST_), we used population means of genomic PCo1 and 2 as a measure of genetic differentiation among populations. To specifically address multicollinearity, we used conditional RDAs to isolate the individual effects of genetic differentiation and the environment on each coevolutionary trait (e.g., TTX resistance of snakes). For example, we tested for the effect of genomic PCo1 and 2 on snake TTX resistance, conditioned on the effect of environmental PC1 and 2. Then we repeated the analysis in reverse, testing for the effect of environmental PC1 and 2 on TTX resistance, conditioned on genomic PCo1 and PCo2. Finally, we conducted a marginal test that included all explanatory variables (TTX of newts, genomic PCo1 and 2 of snakes, and environmental PC1 and 2) and used forward model selection to generate a marginal model that only includes variables with a significant effect on snake TTX resistance. RDAs were calculated using the “rda” function in the vegan R package, including the functions “anova” for significance testing, “RsquareAdj” for model fit, and “ordiR2step” for forward selection.

Both the MRM and RDA analyses revealed a tight correlation between newt TTX levels and the population structure of newts. To further investigate this relationship, we compared Maximum Likelihood clines of the phenotypic and genetic data from each species along the transect (Szymura and Barton 1986, 1991). For newts, we fit a cline to TTX levels using the mean and variance of the log‐transformed TTX data (μg/cm^2^). For garter snakes, we fit (1) a cline to phenotypic TTX resistance using the 50% MAMU dose and variance from the ln‐transformed MAMU data and (2) a genetic cline to the frequency of TTX‐resistant alleles in each population. Two different TTX‐resistant DIV alleles occur in the Pacific Northwest: Na_V_1.4^V^ is generally found at high frequency in the center of the transect, whereas Na_V_1.4^VA^ occurs predominately in southern populations (Fig. 1). We generated separate clines for each allele (data not shown), but the cline fit to the combined frequency of both TTX‐resistant alleles (Fig. 4) was the most representative of variation along the entire transect.

For each species, we compared clinal variation in coevolutionary traits to variation in neutral SNPs summarized by PCo1 from the genomic PCoA. We also fit clines to the ancestry proportions (Q) from the most likely value of K for each species (K = 2). Both analyses of population structure (PCo1 from the PCoA and K = 2 from STRUCTURE) produced concordant results (Fig. S3; Table S6), so only the analysis of PCo1 is presented in the main text. PCoAs have no explicit population genetic assumptions, whereas STRUCTURE assumes that all loci are unlinked at linkage equilibrium and in HWE. Because the PCoA and STRUCTURE analyses produced similar results despite different underlying assumptions, we are confident that the cline‐fitting analyses provided a representative depiction of population structure in each species. Finally, we reran cline‐fitting analyses with the Elk River population removed from the newt dataset, because the PCoA (Fig. 3) and STRUCTURE plot of K = 3 (Fig. S4) both suggest the population is genetically distinct from all others. Similarly, we reran analyses with the Elk River and Lake Tahkenitch localities removed from the snake dataset, because phenotypic TTX resistance (50% MAMU) declines to intermediate levels at the southern end of the cline (Figs. 1 and 4). These reduced cline analyses produced qualitatively similar results, so only the full analyses are presented herein. In fact, when Elk River and Lake Tahkenitch were removed from the snake dataset, congruence between phenotypic clines of newt TTX and snake resistance was even stronger than the results presented below.

We fit clines using the HZAR package in R (Derryberry et al. 2014). We calculated distances along the cline as kilometers (km) from the northernmost sampling site (Clallam). We ran 15 separate models that varied in the number of cline shape parameters estimated. All models estimated the cline center (distance from sampling location 1, *c*) and width (1/maximum slope, *w*), but could additionally estimate combinations of exponential decay curve (tail) parameters (neither tail, right tail only, left tail only, mirrored tails, or both tails separately), which represent the distance from the cline center to the tail (δ) and the slope of the tail (τ). The genetic models varied as to whether they estimated allele frequencies at the cline ends (*p*_min_ and *p*_max_) or fixed them at 0 and 1. All models were then compared using AIC corrected for small sample sizes (AICc) and maximum likelihood parameters were extracted for the best‐fitting model. We considered cline centers with non‐overlapping two log‐likelihood unit support limits (confidence intervals; CIs) to occur in significantly different geographic locations (Baldassarre et al. 2014; Scordato et al. 2017).

## Results and Discussion

### MATCHED TRAIT VARIATION SUPPORTS LOCAL COADAPTATION IN THE ARMS RACE

Geographic patterns of snake resistance and newt TTX were broadly consistent with previous work indicating arms race coevolution has led to correlated phenotypes in each species (Brodie et al. 2002; Hanifin et al. 2008). TTX resistance (50% MAMU dose) of snakes varied among populations along the latitudinal transect (according to non‐overlapping 95% confidence intervals; Fig. 1; Table S1), and phenotypic resistance covaried with the presence of TTX‐resistant alleles in the Na_V_1.4 channel, such that population differentiation in phenotypic resistance was correlated with pairwise F_ST_ differentiation at the DIV p‐loop (MRM, coefficient = 0.964, *p* = 0.032). The TTX levels of newts (μg/cm^2^) also varied among populations (ANOVA; F_8,114_ = 37.43, *p* < 0.001) and sexes (F_1,114_ = 4.37, *p* = 0.039) along the transect (Fig. 1; Table S1). Newt TTX levels were predicted by snake TTX resistance along the transect according to both the MRM (*p* = 0.019; Table 1) and RDA analyses (*p* = 0.025; Table 2).

**Table 1:** Results from multiple regression of distance matrices (MRMs) comparing population differentiation in phenotypic, genetic, and environmental data. Significant *p*‐values are highlighted in bold

Response Variable	Explanatory Variable(s)	Coefficient	*p*‐Value	*R* ^2^
Snake TTX Resistance	Newt TTX	0.662	**0.018**	0.274
	Snake F_ST_	1.442	0.721	0.006
	Environmental PC1	0.025	0.58	0.010
	Environmental PC2	−0.038	0.62	0.018
	Newt TTX + Snake F_ST_			0.338
	Newt TTX	0.873	**0.013**	
	Snake F_ST_	−5.830	0.203	
	Newt TTX + Environmental PC1			0.378
	Newt TTX	1.019	**0.005**	
	Environmental PC1	−0.104	0.051	
	Snake F_ST_ + Environmental PC1			0.012
	Snake F_ST_	0.731	0.884	
	Environmental PC1	0.021	0.708	
Newt TTX	Snake TTX Resistance	0.415	**0.019**	0.274
	Newt F_ST_	5.998	**0.002**	0.414
	Environmental PC1	0.126	**0.001**	0.434
	Environmental PC2	0.017	0.778	0.006
	Snake TTX Resistance + Newt F_ST_			0.501
	Snake TTX Resistance	0.253	0.065	
	Newt F_ST_	4.827	**0.006**	
	Snake TTX Resistance + Environmental PC1			0.644
	Snake TTX Resistance	0.364	**0.01**	
	Environmental PC1	0.117	**0.001**	
	Newt F_ST_ + Environmental PC1			0.623
	Newt F_ST_	4.347	**0.008**	
	Environmental PC1	0.094	**0.008**	

**Table 2:** Results from redundancy analyses (RDAs) assessing how natural enemies, population structure, and environmental conditions each contribute to variation in coevolutionary traits. Conditional RDAs show the proportion of variance explained (prop. var.) by a given explanatory variable after removing the effects of the conditioned variables listed within the parentheses of Condition(). Marginal models only include significant variables after forward model selection. Significant *p*‐values are highlighted in bold

Response Variable	Explanatory Variable(s)	F	*p*‐Value	Prop. var.
Snake TTX Resistance	Newt TTX	8.866	**0.023**	0.496
	Snake Genomic PCos	4.244	0.064	0.448
	Environmental PCs	0.751	0.51	−0.067
	Newt TTX + Condition(Snake Genomic PCos)	1.891	0.242	0.071
	Snake Genomic PCos + Condition(Newt TTX)	1.171	0.389	0.023
	Newt TTX + Condition(Environmental PCs)	14.137	**0.005**	0.732
	Environmental PCs + Condition(Newt TTX)	2.778	0.145	0.170
	Snake Genomic PCos + Condition(Environmental PCs)	3.195	0.136	0.451
	Environmental PCs + Condition(Snake Genomic PCos)	0.690	0.558	−0.064
	**Marginal Model**: Newt TTX	8.866	**0.023**	0.496
Newt TTX	Snake TTX Resistance	8.866	**0.025**	0.496
	Newt Genomic PCos	24.975	**0.003**	0.857
	Environmental PCs	9.859	**0.008**	0.689
	Snake TTX Resistance + Condition(Newt Genomic PCos)	0.052	0.818	−0.027
	Newt Genomic PCos + Condition(Snake TTX Resistance)	7.892	**0.021**	0.334
	Snake TTX Resistance + Condition(Environmental PCs)	14.137	**0.01**	0.214
	Environmental PCs + Condition(Snake TTX Resistance)	15.596	**0.007**	0.407
	Newt Genomic PCos + Condition(Environmental PCs)	4.831	0.076	0.174
	Environmental PCs + Condition(Newt Genomic PCos)	1.140	0.426	0.006
	**Marginal Model**: Newt Genomic PCo1	36.278	**0.001**	0.815

**Figure 2:**
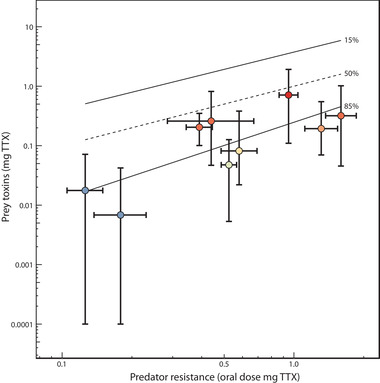
**Functional overlap in levels of predator resistance and prey toxin**. The functional relationship of average adult newt total skin TTX (mg, log scale) is plotted against the oral dose of TTX (mg, log scale) required to reduce the speed of an average adult garter snake to 50% of baseline speed post‐ingestion of TTX for each locality. Individual points are colorized by the average level of newt TTX as in Fig. 1C. Vertical bars represent the full range of newt TTX values observed for each population; horizontal bars represent the 95% confidence interval for the oral 50% dose of the corresponding snake population. The dashed 50% line represents a functional match between newt TTX and snake resistance: the dose of TTX in a newt that would reduce a snake of a given resistance to 50% of its performance. The solid 15% and 85% lines (calculated as best‐fit regressions for each locality) delimit the range of functionally relevant TTX doses for snakes across the range of sampled localities. Below the 85% line, values of TTX resistance are high enough for snakes to consume co‐occurring newts with no reduction in performance or fitness. Above the 15% line, doses of newt TTX are so high that any snake that ingests a newt would be completely incapacitated or killed. Localities with error bars that fall entirely outside the boundaries of these lines (none of which were observed) are considered mismatched and regions where reciprocal selection could not occur.

Quantitative estimates of predator resistance and prey toxin at each locality showed functional overlap in the armaments of each species, implying that locally varying reciprocal selection could occur throughout the geographic mosaic (Fig. 2; Hanifin et al. 2008). The 15 and 85% doses delimit the range of functional overlap between resistance and toxin, outside of which predator and prey are considered so mismatched that variable fitness outcomes and reciprocal selection are unlikely to occur (Williams et al. 2003, 2010; Hanifin et al. 2008). Under this framework, each locality we sampled exhibited some degree of overlap in the phenotypic distributions of snake resistance and newt TTX, indicating potential for reciprocal selection between predator and prey. Interestingly, all comparisons between point estimates of snake resistance and newt TTX fell below the 50% line, suggesting that garter snakes on average tend to have higher levels of resistance than the toxins of co‐occurring newts, a pattern consistent with the prediction that predators experience intense selection when prey are deadly (see below; Brodie and Brodie 1999a). Nonetheless, this analysis suggests that matched trait variation in the geographic mosaic of coevolution could be a result of reciprocal selection.

### PREDATOR AND PREY POPULATIONS DIFFER IN GEOGRAPHIC STRUCTURE

Global F_ST_ values for snakes (F_ST_ = 0.070, 95% CI [0.067, 0.075]) and newts (F_ST_ = 0.068, 95% CI [0.065, 0.069]) were similar, and both species exhibited a strong correlation between pairwise F_ST_ and geographic distance indicative of IBD (dbRDA; snakes, F_2,6_ = 12.02, *p* = 0.001; newts, F_2,6_ = 49.27, *p* = 0.002; Fig. S1). This pattern is consistent with previous work suggesting limited population structure and a recent northward post‐glacial expansion of both species (Janzen et al. 2002; Ridenhour et al. 2007; Hague et al. 2016). In contrast, we found no evidence of IBE for either species (Fig. S1). When controlling for the effect of IBD in conditional dbRDAs, environmental PC1 and 2 did not predict pairwise F_ST_ for snakes (F_2,4_ = 1.09, *p* = 0.429) or newts (F_2,4_ = 0.25, *p* = 0.828).

**Figure 3:**
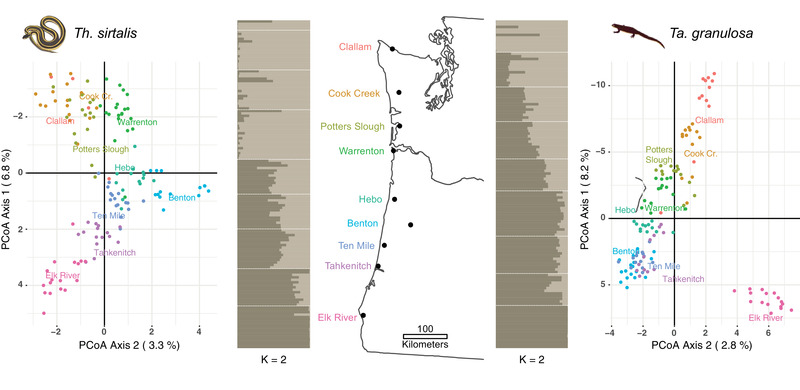
**Populations of predator and prey differ in geographic structure**. Results from the principal coordinate (PCoA) and STRUCTURE analyses of neutral SNPs from snakes and newts. PCoA graphs are rotated 90° to emphasize the major axis of variation corresponding to latitude. The PCo1 values for each individual were used as a neutral expectation in the cline‐fitting analyses. STRUCTURE plots are arranged latitudinally by population, in the same order as the map. Each horizontal bar represents the ancestry assignment of an individual, with populations separated by white dashed lines.

Pairwise estimates of F_ST_ revealed subtle differences in the geographic population structure of predator and prey (Table S4), a pattern that was also supported by the PCoA and Bayesian clustering (STRUCTURE) analyses (Fig. 3). In particular, the major axis of variation from the PCoA (PCo1) and STRUCTURE revealed limited structure among newt populations along the transect, whereas garter snakes showed evidence of population subdivision near the Washington‐Oregon border. For example, the STRUCTURE plot for snakes indicates that subdivision between the two most likely genetic clusters (K = 2) occurs just south of the Columbia River near the Warrenton locality in Oregon. Because pairwise F_ST_ estimates, the PCoAs, and STRUCTURE produced very similar results, yet rely on different assumptions, we are confident in our characterization of population structure for each species. Finally, we used these data to test whether genetic differentiation and local environmental conditions help explain mosaic variation in predator and prey traits.

### MOSAIC TTX VARIATION IS PREDICTED BY NEWT POPULATION STRUCTURE AND THE ENVIRONMENT

Resistance of garter snakes was strictly predicted by prey toxin levels. Newt TTX was the only significant explanatory variable in the MRM (*p* = 0.018; Table 1) and RDA analyses (*p* = 0.023; Table 2). After model selection, the marginal RDA contained newt TTX as the only explanatory variable, which accounted for 49.6% of the variance in snake TTX resistance. In contrast, levels of newt TTX were strongly predicted by population structure, suggesting that reciprocal coadaptation is not fully responsible for mosaic variation in prey toxins. Differentiation among newt populations predicted TTX levels even after controlling for the effect of snake TTX resistance in the multivariate MRM (*p* = 0.006) and the conditional RDA (*p* = 0.021). In fact, after model selection, the marginal RDA included genomic PCo1 as the only explanatory variable, accounting for 81.5% of the variance in newt TTX. The environmental PCs also predicted variation in newt TTX after controlling for snake TTX resistance in the multivariate MRM (*p* = 0.001) and conditional RDA (*p* = 0.007), although this may be driven by multicollinearity of latitudinal explanatory variables (see below). Overall, our results suggest that non‐adaptive factors—especially the population structure of newts—are key correlates of population differences in prey toxin levels, which is not the case for predator resistance. Interestingly, the population structure of newts not only predicted differentiation in newt TTX, but also snake resistance in an MRM (coefficient = 4.632, *p* = 0.037) and an RDA (F_2,6_ = 8.853, *p* = 0.01).

**Figure 4:**
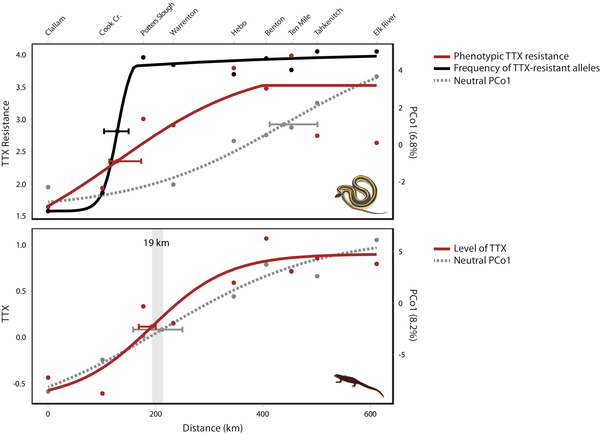
**Levels of prey toxin are strongly predicted by population genetic structure**. Cline‐fitting results for phenotypic and genetic variation are shown, with error bars indicating confidence intervals surrounding the geographic cline centers. Phenotypic clines of TTX resistance in snakes (ln[MAMU + 1]) and TTX levels in newts (log[TTX μg/cm^2^ + 0.1]) are shown in red. For snakes, the frequency of TTX‐resistant alleles in the Na_V_1.4 channel was also modeled (in black). Gray dashed lines represent the neutral expectation of population genomic structure, based on the PCoA. The cline center points of TTX levels and neutral PCo1 for newts are located within in 19 km of each other along the 611 km transect.

We used cline analyses to further evaluate the relationship between phenotypic and genomic variation along the transect. Clinal variation in levels of predator resistance and prey toxins are tightly matched along the 611 km transect; the geographic center points of each cline are located just 64 km apart and do not differ statistically (Fig. 4, Table S6). The cline center of TTX‐resistant alleles in snakes is also located nearby, although it differed statistically from the center of newt TTX. The snake clines representing phenotypic resistance and TTX‐resistant alleles both seem to track levels of prey toxin, deviating from selectively neutral expectations of population structure based on the major axis of variation from the PCoA (PCo1; Fig. 4) and STRUCTURE (Fig. S3). For example, the center points of the phenotypic resistance and genomic PCo1 clines were located a distant 310 km apart. In contrast, clinal variation in TTX levels of newts is highly congruent with population genomic structure based on PCo1 (Fig. 4) and STRUCTURE (Fig. S3). The center points of the TTX and genomic PCo1 clines were located only 19 km apart along the 611 km transect. These two cline centers were also located within 84 km of the centers points for phenotypic resistance and TTX‐resistant alleles in snakes. This striking geographic congruence between newt PCo1, newt TTX, and snake TTX resistance suggests that the population structure of newts is an important determinant of differentiation in prey toxin levels, which in turn influences local levels of predator resistance.

The analyses above imply that TTX levels are strongly predicted by the underlying pattern of IBD among newt populations. The MRM and RDA analyses also suggest that local environmental conditions (encapsulated in PC1) contribute to variation in newt TTX; however, the collinearity of environmental PC1 and newt population differentiation makes it challenging to disentangle the individual contributions of each explanatory variable to TTX levels (Fig. S2). Notably, the environmental PCs were not included in the marginal RDA after model selection (Table 2). Environmental PCs were also not predictive of newt TTX after conditioning for the effects of genomic PCo1 and 2 (*p* = 0.426), whereas the genomic PCos were marginally predictive of TTX after conditioning on the environmental PCs (*p* = 0.076; Table 2). We cannot rule out the importance of the environment, but it seems plausible that the relationship between environmental PC1 and newt TTX could be explained by strong IBD among newt populations along a transect that covaries with latitudinal changes in the environment.

### ASYMMETRIES IN THE ARMS RACE

At first glance, levels of predator resistance and prey toxins are roughly matched across the landscape, but this pattern does not appear to solely result from local arms race coadaptation. Although predator resistance is geographically structured by a signature of local adaptation to prey phenotypes, levels of the prey toxin are strongly predicted by population genetic structure. These unexpected results imply that local variation in levels of the newt toxin across the geographic mosaic can largely be explained by processes unrelated to reciprocal selection, such as historical biogeography, gene flow, and environmental heterogeneity. For example, newt TTX levels are tightly correlated with genetic differentiation and geographic distance along the transect (Fig. S2), exhibiting the signature of IBD among newt populations in the Pacific Northwest (Ridenhour et al. 2007; Hague et al. 2016).

The asymmetric signal of adaptation we observed in predator and prey may reflect differences in the mechanisms that underlie phenotypic variation in each species. TTX resistance in garter snakes is largely due to a small number of amino acid changes to the DIV p‐loop of the Na_V_1.4 channel (Geffeney et al. 2005; Feldman et al. 2010; Hague et al. 2017). These large‐effect mutations may permit rapid evolution of TTX resistance in the arms race. On the other hand, genes associated with TTX biosynthesis in newts have yet to be discovered; however, toxin production likely requires a complicated biosynthetic pathway. For example, biosynthesis of a similar neurotoxin found in pufferfish, saxitoxin (STX), involves gene expression in a cluster of up to 26 genes (Moczydlowski 2013). A complex genetic basis for TTX in newts (e.g., many unlinked loci of small effect) could make TTX evolution more sensitive to the homogenizing effects of gene flow (Lenormand 2002), which may explain the tight correlation between TTX levels and genetic differentiation among newt populations.

An evolutionary response in newts could also be obscured by environmental effects that disproportionally contribute to variance in TTX levels compared to resistance of snakes. Some researchers suggest exogenous factors, like environmentally‐derived precursors, may affect the ability of newts to synthesize or sequester TTX (Yotsu et al. 1990; Yasumoto and Yotsu‐Yamashita 1996). TTX‐bearing marine taxa are generally thought to obtain toxins through the food chain or a bacterial symbiont, and high individual and regional variation of TTX levels in pufferfish is cited as evidence of an exogenous source of the toxin (Kanoh 1988; Noguchi and Arakawa 2008; also see Vaelli et al. 2020). Barring issues of multicollinearity, latitudinal variation in temperature and precipitation was predictive of newt TTX levels after controlling for the effect of snake TTX resistance (Tables 1 and 2), suggesting that TTX production may partially depend on unknown environmental factors.

Finally, asymmetries could arise from a selective imbalance associated with the interactions between predator and prey. In antagonistic interactions, the species under more intense selection is generally expected to be better adapted to local conditions (Gandon 2002). While prey are typically thought to experience stronger selection than their predators (the “life‐dinner principle”) (Dawkins and Krebs 1979), this asymmetry may be reversed when prey contain deadly toxins like TTX (Brodie and Brodie 1999a). In fact, populations in central Oregon are the most toxic newts known (Hanifin et al. 2008), so non‐resistant predators should experience severe fitness consequences. Moreover, we found that garter snakes tend to have greater functional estimates of resistance than the levels of TTX in co‐occurring newts (Figure 2), implying intense selection and a stronger evolutionary response by predators.

### IMPLICATIONS FOR THE GEOGRAPHIC MOSAIC OF ARMS RACE COEVOLUTION

Non‐adaptive processes like gene flow and environmental heterogeneity may explain local patterns of variation in newt TTX across the geographic mosaic, but the extreme levels of prey toxin and predator resistance at phenotypic hotspots such as central Oregon are likely a result of arms race coevolution. Taken together, the overall pattern suggests that reciprocal selection and coadaptation can explain escalated TTX levels in specific hotspots of coevolution, but global variation in TTX across the geographic mosaic is dictated by other processes. For example, TTX may be favored in specific hotspots like southern Oregon, but not in surrounding regions like northern Washington (i.e., “coldspots”), and the tight congruence between clinal variation in newt TTX and neutral SNPs reflects the homogenizing effects of gene flow between hot‐ and coldspots (Fig. 4).

The notable importance of prey population structure in the geographic mosaic of coevolution points to an influential role of “trait remixing”, a largely untested process in the geographic mosaic theory thought to generate spatial variation at the phenotypic interface of coevolution (Thompson 2005; Gomulkiewicz et al. 2007). The neutral processes of drift and gene flow are predicted to continually alter the spatial distribution of allelic and phenotypic variation, potentially interfering with local selection. Gene flow outwards from hotspots of coevolution is predicted to alter dynamics in surrounding populations (Gomulkiewicz et al. 2000; Thompson et al. 2002), and if gene flow is high, the population with the strongest reciprocal effects on fitness is expected to dictate broader landscape patterns of trait variation (Gomulkiewicz et al. 2000; Gandon 2002; Gandon and Michalakis 2002). The homogenizing effects of gene flow may be less influential in snake populations due to the simple genetic basis of TTX resistance and/or strong selection on predators.

Our results highlight that not all landscape patterns of phenotypic matching in natural enemies are necessarily the result of coevolution and a mosaic of reciprocal selection (Gomulkiewicz et al. 2007). External factors such as population structure (Dybdahl and Lively 1996), environmental conditions (Toju and Sota 2005), evolutionary constraints (Hague et al. 2018), or interactions with other species (Benkman et al. 2001) are likely to have unique effects on the evolution of each species. In the geographic mosaic of arms race coevolution, predator and prey exhibit matched trait variation across the landscape, yet phenotypic differentiation in newt toxins is largely predicted by population structure and the environment. At face value, matched weaponry between natural enemies seems to demonstrate the power of reciprocal selection, but adaptation at the phenotypic interface of coevolution is almost certain to be asymmetric. Coevolution occurs within the broader evolutionary context of each species and non‐adaptive processes may ultimately bound the potential contribution of reciprocal selection to the geographic mosaic of coevolution.

## CONFLICT OF INTEREST

The authors declare no conflict of interest.

## AUTHOR CONTRIBUTIONS

MTJH designed the project, collected specimens, generated genetic data, and performed statistical analyses. ANS collected phenotypic data on newt TTX levels. CRF collected specimens and phenotypic data on snake resistance. EDB, Jr. collected phenotypic data on snake resistance and provided leadership on the project. EDB III designed the project and provided leadership. All authors prepared the manuscript.

## DATA ARCHIVING

The ddRADseq data will be made available on GenBank upon manuscript acceptance. DIV p‐loop sequences are available on GenBank (MT043460‐MT043727). All genomic and phenotypic data and the code for statistical analyses are available on Dryad (https://doi.org/10.5061/dryad.51c59zw5k).

Associate Editor: L. Bromham

## Supporting information


**Supplemental Figure S1**. Distance‐based redundancy analyses (dbRDAs) illustrating the relationships among neutral F_ST_ and latitude and longitude (IBD) and neutral F_ST_ and environmental PC1 and 2 (IBE).
**Supplemental Figure S2**. Correlations between each coevolutionary trait and genetic, geographic, and environmental distances.
**Supplemental Figure S3**. Comparison of neutral clines fit to (1) PCo1 values from the PCoA and (2) average ancestry assignment values (K=2) from the STRUCTURE analysis.
**Supplemental Figure S4**. STRUCTURE results for K=2‐4. The most likely number of genetic cluster was K=2 for both species (Figure 2).
**Supplemental Table S1**. Datasets for each sampling location along the latitudinal transect.
**Supplemental Table S2**. For each locality, functional estimates of oral doses of TTX (mg) required to reduce the speed of an average adult *Th. sirtalis* to 15, 50, and 85% of baseline speed post‐ingestion and the total skin TTX dose (mg) in adult *Ta. granulosa*.
**Supplemental Table S3**. Population genetic diversity statistics from neutral SNPs in each species.
**Supplemental Table S4**. Pairwise F_ST_ statistics for the neutral SNP datasets of each species.
**Supplemental Table S5**. PC loadings for the 19 biolclim variables.
**Supplemental Table S6**. Results from cline‐fitting analyses.
