# Supplementary material for: The geographic mosaic of arms race coevolution is closely matched to prey population structure
Source: Evol Lett. 2020 Jun 9;4(4):317–32. doi: 10.1002/evl3.184 (PMC7403720; doi:10.1002/evl3.184)
Supplement: Supplementary file 1 — Supplemental Figure S1. Distance‐based redundancy analyses (dbRDAs) illustrating the relationships among neutral FST and latitude and longitude (IBD) and neutral FST and environmental PC1 and 2 (IBE). Supplemental Figure S2. Correlations between each coevolutionary trait and genetic, geographic, and environmental distances. Supplemental Figure S3. Comparison of neutral clines fit to (1) PCo1 values from the PCoA and (2) average ancestry assignment values (K=2) from the STRUCTURE analysis. Supplemental Figure S4. STRUCTURE results for K=2‐4. The most likely number of genetic cluster was K=2 for both species (Figure 2). Supplemental Table S1. Datasets for each sampling location along the latitudinal transect. Supplemental Table S2. For each locality, functional estimates of oral doses of TTX (mg) required to reduce the speed of an average adult Th. sirtalis to 15, 50, and 85% of baseline speed post‐ingestion and the total skin TTX dose (mg) in adult Ta. granulosa. Supplemental Table S3. Population genetic diversity statistics from neutral SNPs in each species. Supplemental Table S4. Pairwise FST statistics for the neutral SNP datasets of each species. Supplemental Table S5. PC loadings for the 19 biolclim variables. Supplemental Table S6. Results from cline‐fitting analyses. [file EVL3-4-317-s001.docx]

**SUPPLEMENTAL INFORMATION**

**
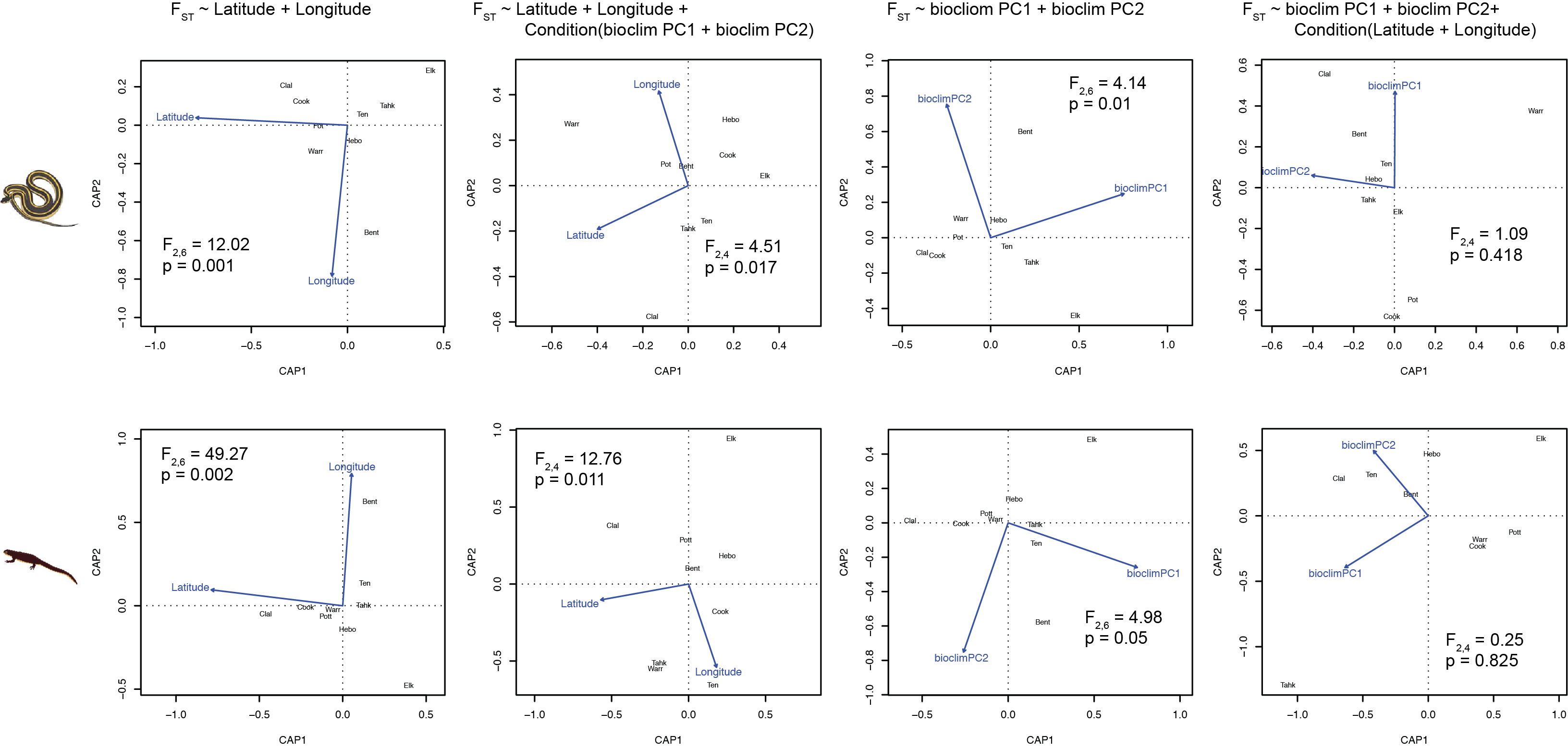
**

**Supplemental Figure S1.** Distance-based redundancy analyses (dbRDAs) illustrating the relationships among neutral F_ST_ and latitude and longitude (IBD) and neutral F_ST_ and environmental PC1 and 2 (IBE). Conditional dbRDAs show the relationship between F_ST_ and the given explanatory variables after removing the effects of the conditioned variables listed within the parentheses of Condition(). For both snakes and newts, we found evidence for IBD in conditional dbRDAs that remove the effect of environmental PC1 and 2. In contrast, we found no evidence for IBE after removing the effect of latitude and longitude.


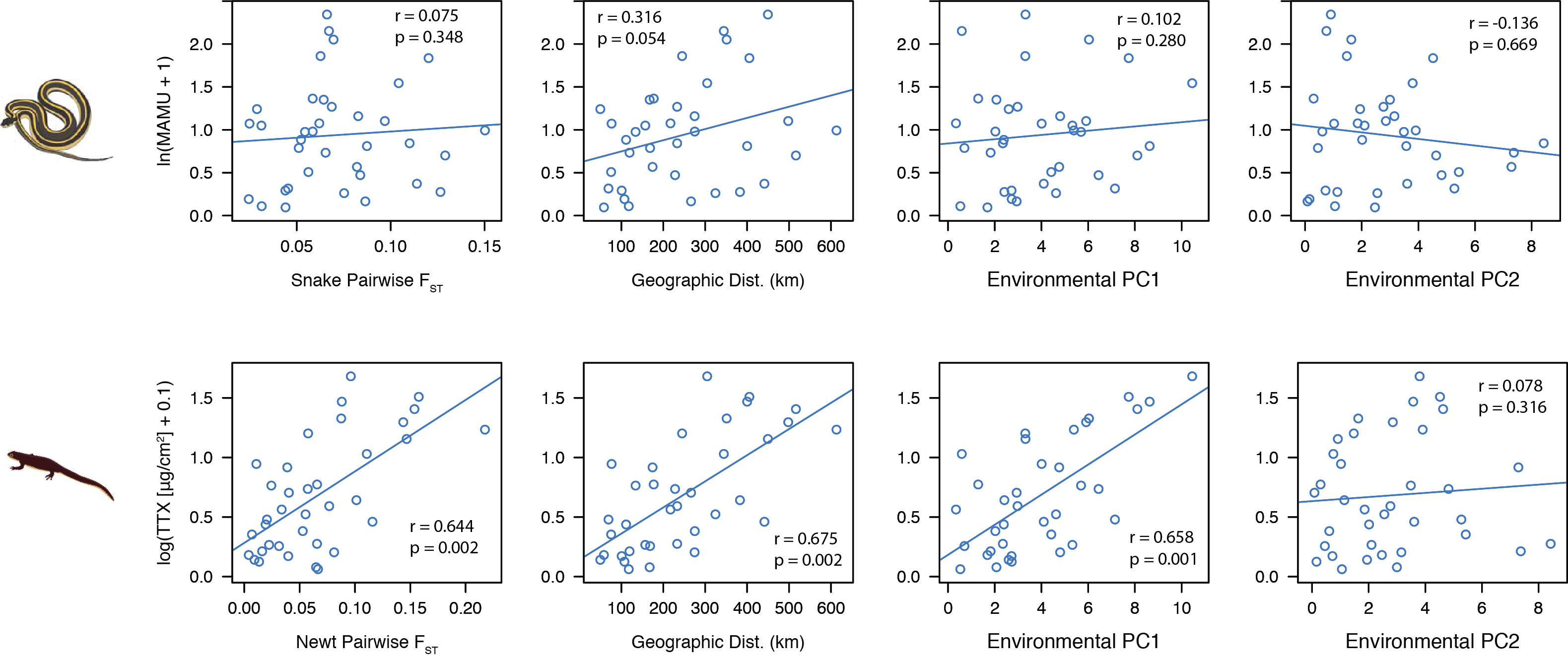


**Supplemental Figure S2.** Correlations between each coevolutionary trait and genetic, geographic, and environmental distances. Correlations were assessed using Mantel tests as described in the main text.

**
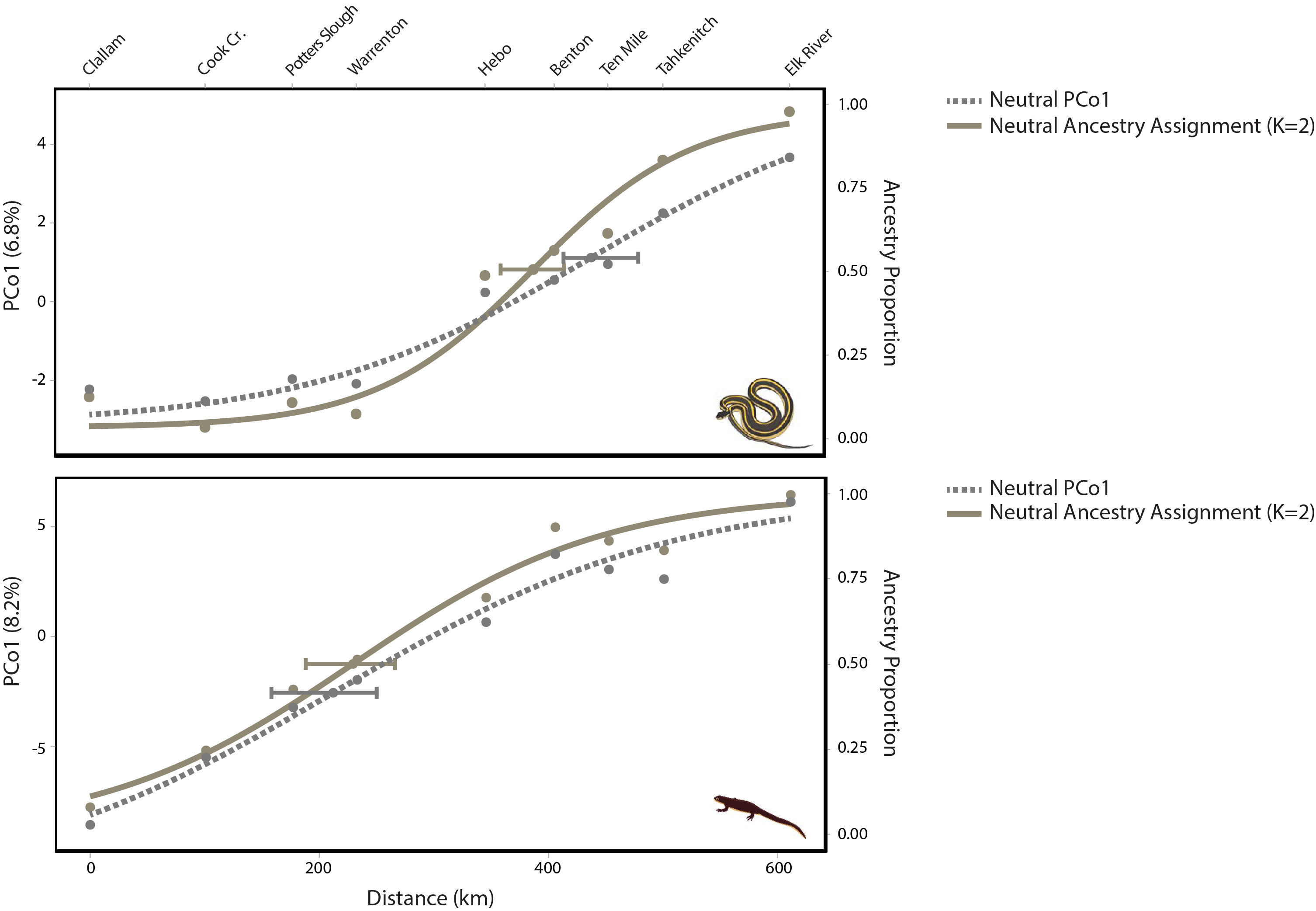
**

**Supplemental Figure S3.** Comparison of neutral clines fit to (1) PCo1 values from the PCoA and (2) average ancestry assignment values (K=2) from the STRUCTURE analysis. Error bars indicate confidence intervals surrounding the geographic cline centers.

**
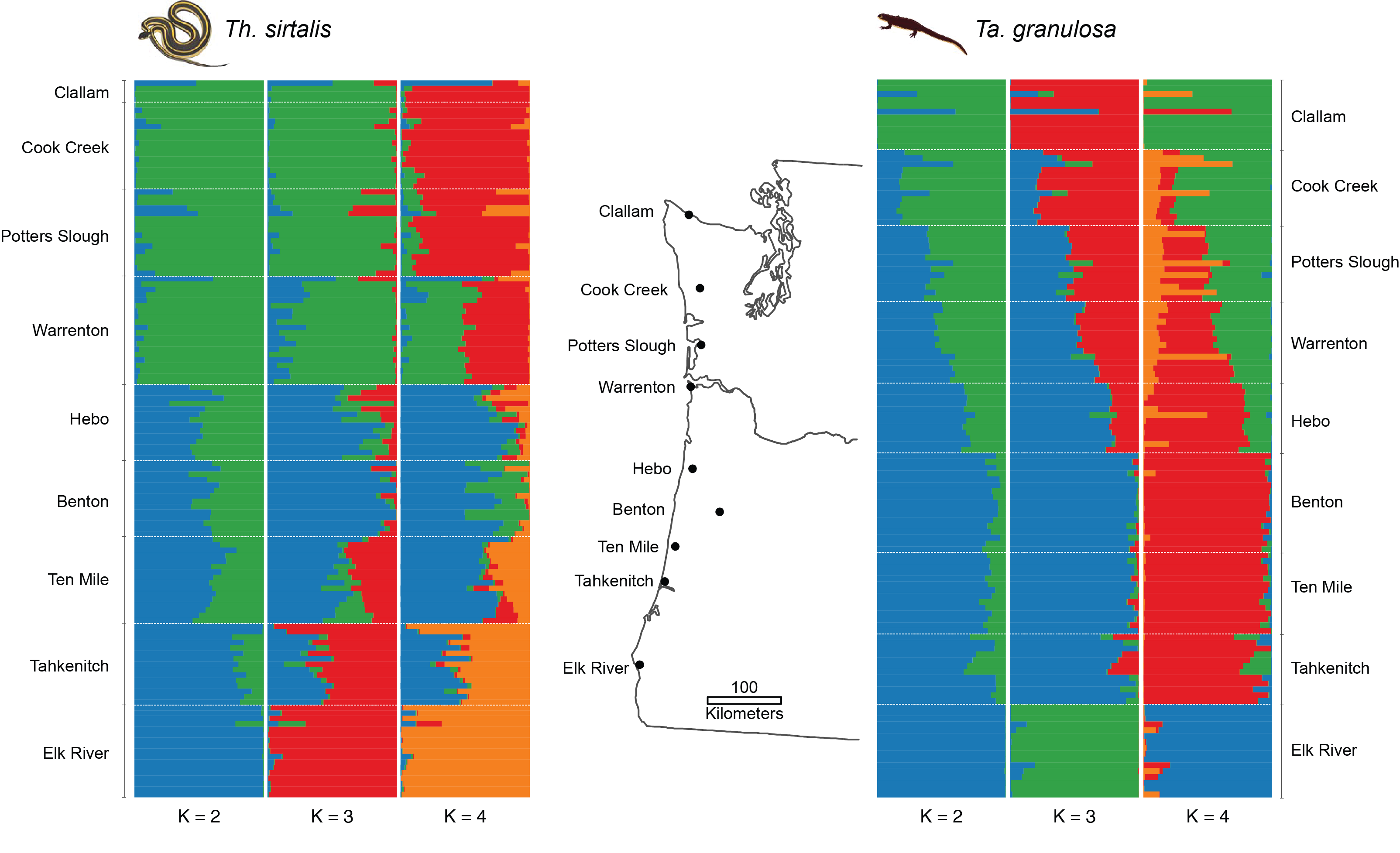
**

**Supplemental Figure S4.** STRUCTURE results for K=2-4. The most likely number of genetic cluster was K=2 for both species (Figure 2). STRUCTURE plots are arranged latitudinally by population, in the same order as the map. Each horizontal bar represents the ancestry assignment of an individual, with populations separated by white dashed lines.

**Supplemental Table S1.** Datasets for each sampling location along the latitudinal transect. The total number of animals sampled in this study is shown first, followed by the number of individuals (*n*) included in each analysis. For estimates of phenotypic resistance in *Th. sirtalis*, we combined individuals from this study with previously published racetrack data from the same sampling locations (Brodie et al. 2002; Ridenhour 2004). In the DIV p-loop, the number (*n*) of alleles sampled accounts for females, the hemizygous sex with only one genetic copy of Na_V_1.4. Results are shown for the joint test of Hardy-Weinberg Equilibrium (HWE) and Equality of Allele Frequencies (EAF) from Gendreau et al. (2020).

|  |  | **Total animals sampled in this study** | |  | ***Th. sirtalis*** | | | | | |  | ***Ta. granulosa*** | | | |
| --- | --- | --- | --- | --- | --- | --- | --- | --- | --- | --- | --- | --- | --- | --- | --- |
|  |  |  |  |  | **Phenotypic TTX resistance** | | | **DIV p-loop** | | **ddRADseq** |  | **TTX** | | | **ddRADseq** |
| **Location** | **County** | ***Th. sirtalis*** | ***Ta. granulosa*** |  | ***n*** | **50% MAMU** | **SE** | ***n* alleles** | **HWE & EAF test**  **p-value** | ***n*** |  | ***n*** | **mean (ug/cm^2^)** | **SE** | ***n*** |
| Clallam | Clallam, WA | 11 | 12 |  | 53 | 4.163 | 0.076 | 14 | NA | 4 |  | 12 | 0.731 | 0.307 | 12 |
| Cook Creek | Grays Harbor, WA | 17 | 14 |  | 14 | 5.915 | 0.119 | 26 | 1.000 | 16 |  | 14 | 0.236 | 0.112 | 13 |
| Potter's Slough | Pacific, WA | 20 | 15 |  | 42 | 19.203 | 0.090 | 27 | 1.000 | 16 |  | 15 | 2.75 | 0.686 | 13 |
| Warrenton | Clatsop, OR | 24 | 15 |  | 323 | 17.357 | 0.038 | 36 | 0.257 | 20 |  | 14 | 1.63 | 0.241 | 14 |
| Hebo | Tillamook, OR | 15 | 15 |  | 2 | 43.413 | 0.085 | 21 | 0.708 | 14 |  | 15 | 4.65 | 0.822 | 12 |
| Benton | Benton, OR | 20 | 18 |  | 386 | 31.380 | 0.047 | 23 | 0.347 | 14 |  | 18 | 16.9 | 3.1 | 17 |
| Ten Mile | Lane, OR | 16 | 15 |  | 93 | 52.817 | 0.079 | 26 | 0.451 | 16 |  | 14 | 7.43 | 1.85 | 14 |
| Lake Tahkenitch | Douglas, OR | 29 | 14 |  | 17 | 14.552 | 0.227 | 42 | 0.182 | 15 |  | 13 | 10.7 | 2.56 | 12 |
| Elk River | Curry, OR | 17 | 20 |  | 43 | 12.935 | 0.063 | 20 | 0.324 | 17 |  | 15 | 6.52 | 0.612 | 16 |
|  | **Total** | 169 | 138 |  | 932 |  |  | 235 |  | 132 |  | 130 |  |  | 123 |

**Supplemental Table S2.** For each locality, functional estimates of oral doses of TTX (mg) required to reduce the speed of an average adult *Th. sirtalis* to 15, 50, and 85% of baseline speed post-ingestion and the total skin TTX dose (mg) in adult *Ta. granulosa*.

|  | ***Th. sirtalis*** | | |  | ***Ta. granulosa*** | | |
| --- | --- | --- | --- | --- | --- | --- | --- |
| **Population** | **50% oral dose (mg)** | **15% oral dose (mg)** | **85% oral dose (mg)** |  | **mean TTX (mg)** | **max TTX (mg)** | **min TTX (mg)** |
| Clallam | 0.1257 | 0.5710 | 0.0102 |  | 0.0176 | 0.0715 | 0.0001 |
| Cook Creek | 0.1787 | 0.4614 | 0.0585 |  | 0.0068 | 0.0422 | 0.0001 |
| Potter's Slough | 0.5800 | 1.9169 | 0.1610 |  | 0.0816 | 0.3813 | 0.0220 |
| Warrenton | 0.5243 | 2.2042 | 0.1074 |  | 0.0475 | 0.1263 | 0.0053 |
| Hebo | 1.3113 | 1.7279 | 0.9935 |  | 0.1933 | 0.5495 | 0.0698 |
| Benton | 0.9479 | 3.9647 | 0.2093 |  | 0.7107 | 1.9188 | 0.1097 |
| Ten Mile | 1.5954 | 11.1998 | 0.2051 |  | 0.3188 | 1.0166 | 0.0455 |
| Lake Tahkenitch | 0.4396 | 3.4492 | 0.0332 |  | 0.2590 | 0.8172 | 0.0468 |
| Elk River | 0.3907 | 1.4213 | 0.0918 |  | 0.2043 | 0.3495 | 0.1007 |

**Supplemental Table S3.** Population genetic diversity statistics from neutral SNPs in each species. Sample size (*n*), average observed heterozygosity (H_O_), expected heterozygosity (H_E_), and nucleotide diversity (π) are shown, along with standard deviations (SD).

|  | ***Th. sirtalis*** | | | | | | |  | ***Ta. granulosa*** | | | | | | |
| --- | --- | --- | --- | --- | --- | --- | --- | --- | --- | --- | --- | --- | --- | --- | --- |
| **Population** | ***n*** | **H_O_** | **H_O_ SD** | **H_E_** | **H_E_ SD** | π | π **SD** |  | ***n*** | **H_O_** | **H_O_ SD** | **H_E_** | **H_E_ SD** | π | π **SD** |
| Clallam | 4 | 0.328 | 0.297 | 0.294 | 0.218 | 0.268 | 0.146 |  | 12 | 0.278 | 0.248 | 0.253 | 0.196 | 0.203 | 0.100 |
| Cook Creek | 16 | 0.311 | 0.231 | 0.288 | 0.177 | 0.264 | 0.129 |  | 13 | 0.324 | 0.237 | 0.294 | 0.178 | 0.218 | 0.107 |
| Potters Slough | 16 | 0.319 | 0.230 | 0.293 | 0.169 | 0.257 | 0.126 |  | 13 | 0.352 | 0.225 | 0.320 | 0.163 | 0.255 | 0.125 |
| Warrenton | 20 | 0.297 | 0.218 | 0.292 | 0.170 | 0.257 | 0.125 |  | 14 | 0.321 | 0.200 | 0.323 | 0.156 | 0.263 | 0.129 |
| Hebo | 14 | 0.292 | 0.218 | 0.302 | 0.168 | 0.275 | 0.135 |  | 12 | 0.330 | 0.205 | 0.332 | 0.159 | 0.252 | 0.124 |
| Benton | 14 | 0.337 | 0.270 | 0.288 | 0.184 | 0.233 | 0.114 |  | 17 | 0.335 | 0.198 | 0.330 | 0.154 | 0.272 | 0.132 |
| Ten Mile | 16 | 0.363 | 0.246 | 0.303 | 0.163 | 0.271 | 0.132 |  | 14 | 0.325 | 0.201 | 0.332 | 0.157 | 0.256 | 0.125 |
| Tahkenitch | 15 | 0.303 | 0.220 | 0.296 | 0.169 | 0.258 | 0.126 |  | 12 | 0.376 | 0.244 | 0.332 | 0.169 | 0.242 | 0.119 |
| Elk River | 17 | 0.306 | 0.232 | 0.285 | 0.180 | 0.248 | 0.121 |  | 16 | 0.300 | 0.204 | 0.318 | 0.174 | 0.230 | 0.112 |
| Total | 132 | 0.317 | 0.240 | 0.293 | 0.178 | 0.259 | 0.013 |  | 123 | 0.327 | 0.218 | 0.315 | 0.168 | 0.243 | 0.022 |

**Supplemental Table S4.** Pairwise F_ST_ statistics for the neutral SNP datasets of each species. For *Th. sirtalis*, F_ST_ differentiation at the DIV p-loop of the Na_V_1.4 channel is also shown. F_ST_ values are shaded red to illustrate the extent of differentiation among different populations (white=0.00, red=1.00).

| ***Th. sirtalis* pairwise F_ST_ of neutral SNPs** | | | | | | | | |
| --- | --- | --- | --- | --- | --- | --- | --- | --- |
|  | **Clallam** | **Cook Creek** | **Potters Slough** | **Warrenton** | **Hebo** | **Benton** | **Ten Mile** | **Tahkenitch** |
| **Cook Creek** | 0.044 |  |  |  |  |  |  |  |
| **Potters Slough** | 0.058 | 0.025 |  |  |  |  |  |  |
| **Warrenton** | 0.069 | 0.054 | 0.044 |  |  |  |  |  |
| **Hebo** | 0.067 | 0.063 | 0.051 | 0.052 |  |  |  |  |
| **Benton** | 0.120 | 0.104 | 0.084 | 0.082 | 0.045 |  |  |  |
| **Ten Mile** | 0.066 | 0.070 | 0.058 | 0.062 | 0.024 | 0.056 |  |  |
| **Tahkenitch** | 0.097 | 0.087 | 0.075 | 0.086 | 0.031 | 0.065 | 0.029 |  |
| **Elk River** | 0.150 | 0.129 | 0.114 | 0.127 | 0.083 | 0.110 | 0.064 | 0.031 |

| ***Th. sirtalis* pairwise F_ST_ of DIV p-loop** | | | | | | | | |
| --- | --- | --- | --- | --- | --- | --- | --- | --- |
|  | **Clallam** | **Cook Creek** | **Potters Slough** | **Warrenton** | **Hebo** | **Benton** | **Ten Mile** | **Tahkenitch** |
| **Clallam** |  |  |  |  |  |  |  |  |
| **Cook Creek** | 0.039 |  |  |  |  |  |  |  |
| **Potters Slough** | 0.688 | 0.625 |  |  |  |  |  |  |
| **Warrenton** | 0.722 | 0.665 | 0.014 |  |  |  |  |  |
| **Hebo** | 0.575 | 0.497 | 0.034 | 0.154 |  |  |  |  |
| **Benton** | 0.652 | 0.579 | 0.025 | 0.162 | -0.034 |  |  |  |
| **Ten Mile** | 0.593 | 0.514 | 0.105 | 0.251 | -0.028 | -0.019 |  |  |
| **Tahkenitch** | 0.820 | 0.739 | 0.455 | 0.595 | 0.254 | 0.245 | 0.147 |  |
| **Elk River** | 0.805 | 0.725 | -0.010 | -0.024 | 0.141 | 0.132 | 0.230 | 0.603 |

| ***Ta. granulosa* pairwise F_ST_ of neutral SNPs** | | | | | | | | |
| --- | --- | --- | --- | --- | --- | --- | --- | --- |
|  | **Clallam** | **Cook Creek** | **Potters Slough** | **Warrenton** | **Hebo** | **Benton** | **Ten Mile** | **Tahkenitch** |
| **Clallam** |  |  |  |  |  |  |  |  |
| **Cook Creek** | 0.040 |  |  |  |  |  |  |  |
| **Potters Slough** | 0.066 | 0.011 |  |  |  |  |  |  |
| **Warrenton** | 0.077 | 0.024 | 0.004 |  |  |  |  |  |
| **Hebo** | 0.111 | 0.058 | 0.031 | 0.019 |  |  |  |  |
| **Benton** | 0.158 | 0.096 | 0.057 | 0.039 | 0.020 |  |  |  |
| **Ten Mile** | 0.147 | 0.088 | 0.053 | 0.034 | 0.013 | 0.007 |  |  |
| **Tahkenitch** | 0.144 | 0.088 | 0.055 | 0.040 | 0.022 | 0.016 | 0.009 |  |
| **Elk River** | 0.218 | 0.154 | 0.116 | 0.101 | 0.081 | 0.066 | 0.065 | 0.067 |

**Supplemental Table S5.** PC loadings for the 19 biolclim variables.

| **Bioclim Code** | **Bioclim Variable** | **PC1** | **PC2** |
| --- | --- | --- | --- |
| bio1 | Annual Mean Temperature | 0.286 | -0.051 |
| bio2 | Mean Diurnal Range (Mean of monthly (max temp - min temp)) | 0.170 | 0.270 |
| bio3 | Isothermality (BIO2/BIO7) (×100) | 0.173 | -0.287 |
| bio4 | Temperature Seasonality (standard deviation ×100) | -0.029 | 0.400 |
| bio5 | Max Temperature of Warmest Month | 0.157 | 0.341 |
| bio6 | Min Temperature of Coldest Month | 0.174 | -0.311 |
| bio7 | Temperature Annual Range (BIO5-BIO6) | 0.056 | 0.392 |
| bio8 | Mean Temperature of Wettest Quarter | 0.208 | -0.286 |
| bio9 | Mean Temperature of Driest Quarter | 0.200 | 0.277 |
| bio10 | Mean Temperature of Warmest Quarter | 0.215 | 0.254 |
| bio11 | Mean Temperature of Coldest Quarter | 0.207 | -0.288 |
| bio12 | Annual Precipitation | -0.290 | -0.040 |
| bio13 | Precipitation of Wettest Month | -0.275 | -0.027 |
| bio14 | Precipitation of Driest Month | -0.287 | 0.054 |
| bio15 | Precipitation Seasonality (Coefficient of Variation) | 0.230 | 0.009 |
| bio16 | Precipitation of Wettest Quarter | -0.279 | -0.044 |
| bio17 | Precipitation of Driest Quarter | -0.297 | 0.012 |
| bio18 | Precipitation of Warmest Quarter | -0.297 | 0.025 |
| bio19 | Precipitation of Coldest Quarter | -0.281 | -0.037 |

**Supplemental Table S6.** Results from cline-fitting analyses. The mean value and its geographic center point along the cline are shown for each dataset, in addition to cline width. Confidence intervals (CI) are also listed.

|  |  | **Mean** | **Center (km)** | **Lower CI** | **Upper CI** | **Width (km)** | **Lower CI** | **Upper CI** |
| --- | --- | --- | --- | --- | --- | --- | --- | --- |
| ***Th. sirtalis*** | **50% MAMU dose** | 2.350 | 128.543 | 113.922 | 173.242 | 510.820 | 314.218 | 539.450 |
|  | **Freq. of TTX resistant alleles** | 0.500 | 128.843 | 104.118 | 149.925 | 55.589 | 5.569 | 101.699 |
|  | **Neutral PCo1** | 1.119 | 438.199 | 414.155 | 479.387 | 497.404 | 401.353 | 610.354 |
|  | **STRUCTURE (K=2)** | 0.506 | 387.698 | 359.552 | 415.000 | 276.425 | 206.047 | 368.235 |
| ***Ta. granulosa*** | **TTX** | 0.118 | 193.184 | 168.778 | 200.283 | 284.949 | 207.732 | 363.750 |
|  | **Neutral PCo1** | -2.562 | 212.131 | 158.176 | 250.096 | 599.042 | 532.016 | 611.000 |
|  | **STRUCTURE (K=2)** | 0.500 | 229.646 | 188.078 | 265.566 | 440.284 | 348.296 | 574.741 |
